# Understand the difference between clinical measured ultrafiltrationand real ultrafiltration in peritoneal dialysis

**DOI:** 10.1186/s12882-021-02589-3

**Published:** 2021-11-15

**Authors:** Zanzhe Yu, Zhuqing Wang, Qin Wang, Minfang Zhang, Haijiao Jin, Li Ding, Hao Yan, Jiaying Huang, Yan Jin, Simon Davies, Wei Fang, Zhaohui Ni

**Affiliations:** 1 Department of Nephrology, Renji Hospital, School of Medicine, Shanghai Jiaotong University, Shanghai, China; 2 Faculty of Medicine and Health Sciences, Keele University, Keele, UK

## Abstract

**Background:**

It has been noticed for years that ultrafiltration (UF) is important for survival in peritoneal dialysis. On the other hand, precise and convenient UF measurement suitable for patient daily practice is not as straight forward as it is to measure UF in the lab. Both overfill and flush before fill used to be source of measurement error for clinical practice. However, controversy finding around UF in peritoneal dialysis still exists in some situation. The current study was to understand the difference between clinical measured UF and real UF. The effect of evaporation and specific gravity in clinical UF measurement were tested in the study.

**Methods:**

Four different brands of dialysate were purchased from the market. The freshest dialysate available in the market were intentionally picked. The bags were all 2 L, 2.5% dextrose and traditional lactate buffered PD solution. They were stored in four different conditions with controlled temperature and humidity. The bags were weighted at baseline, 6 months and 12 months of storage. Specific gravity was measured in mixed 24 h drainage dialysate from 261 CAPD patients when they come for their routine solute clearance test.

**Results:**

There was significant difference in dialysate bag weight at baseline between brands. The weight declined significantly after 12 month’s storage. The weight loss was greater in higher temperature and lower humidity. The dialysate in non-PVC package lose less weight than PVC package. The specific gravity of dialysate drainage was significantly higher than pure water and it was related to dialysate protein concentration.

**Conclusion:**

Storage condition and duration, as well as the type of dialysate package have significant impact in dialysate bag weight before use. Evaporation is likely to be the reason behind. The fact that specific gravity of dialysate drainage is higher than 1 g/ml overestimates UF in manual exchanges, which contributes to systemic measurement error of ultrafiltration in CAPD.

**Trial registration:**

ClinicalTrials.gov ID: NCT03864120 (March 8, 2019) (Understand the Difference Between Clinical Measured Ultrafiltration and Real Ultrafiltration).

**Supplementary Information:**

The online version contains supplementary material available at 10.1186/s12882-021-02589-3.

## Introduction

It has been noticed for years that ultrafiltration (UF) is important for survival in peritoneal dialysis. Adequate UF has been part of the guideline target [1–3]. It is also an important parameter of peritoneal membrane function. Incorrect UF measurement may mislead the diagnosis of ultrafiltration failure [4]. Precise measurement of UF is also the base of correct estimation of other solute removal, such as sodium removal and urea and creatinine clearance [5, 6].

Clinical measured UF is different from measuring fluid volume in lab. Convenient and minimal risk of exposure to body fluid, both need to be considered. For these reasons, weight the bags is preferable than measuring volume for manual exchanges. In early days, it was common to neglect overfill, which contributed to systemic UF measurement error [4, 7, 8]. As an example, It is widely accepted that CAPD is as good as APD in terms of preserving residual renal function, if not better. In clinical practice, it is also common to switch patient from CAPD to APD with unsatisfied fluid status. Meanwhile, a favored 24 h UF in CAPD was noticed in several studies at that time [6, 9, 10]. Neglecting overfill used to be the reason to over estimate UF in CAPD [4, 7, 11].

Current clinical UF measurements suggested is to weight the “whole” drained bag and minus the weight of empty bag and the expected input volume, the labeled volume plus overfill volume [5, 8]. It minimizes the work load and the risk of exposing to body fluids for the patients and medical staff. It is a reasonable measurement method for daily practice.

However, controversy finding around UF in peritoneal dialysis still exists to some degree. For example, there were studies which had clearly accounted for overfill still found a favored UF in CAPD compared to APD [12]. The question is whether there is any other issue around clinical UF measurement has not been clarified?

The current study was to understand the difference between clinical measured UF and real UF. The effect of evaporation and specific gravity in clinical UF measurement were tested in the study.

The study adheres to CONSORT 2010 reporting guideline.

## Methods

### Study design and material

Four different brands of dialysate were purchased from the market. The dialysate were all 2 L, 2.5% dextrose and traditional lactate buffered PD solution. Brand A and B were in PVC package. Brand C and D were in non-PVC package (Table 1). The freshest dialysate available in the market were intentionally picked. The time duration between manufacture data to baseline measurement were from 43 to 105 days.

**Table 1:** Features of the four brands bags used in the study

	Glucose (%)	Buffer agent	Ca^++^ (mmol/L)	PVC/non-PVC	Days after manufacture
Brand A (Baxter, Guangzhou)	2.27%	Lactate	1.77	PVC	43
Brand B (Shuanghe, Beijing)	2.27%	Lactate	1.77	PVC	50
Brand C (Huaren, Shanghai)	2.27%	Lactate	1.77	non-PVC	62
Brand D (Fresenius, Jiangsu)	2.27%	Lactate	1.24	non-PVC	105

At baseline, the bags were weighted as whole. The outer package of 4 bags in each brands were removed and weighted separately. The other intact bags were then stored in four different conditions with controlled temperature and humidity. The intact bags were weighted at baseline, 6 months and 12 months of storage. The detailed temperature and humidity of each condition was shown as following.

- Condition 1, 5 °C and uncontrolled humidity. *N* = 5 for brand A, B, D. *N* = 4 for brand C.
- Condition 2, 25 °C and 40% humidity. *N* = 5 for brand A, B, D. *N* = 3 for brand C.
- Condition 3, 30 °C and 30% humidity. *N* = 5 for brand A, B, D. *N* = 3 for brand C.
- Condition 4,40 °C and 20% humidity. *N* = 5 for brand A, B, D. *N* = 4 for brand C.

### Sample size calculation

According to preliminary measurement, a 2 L dialysate bag was weighted around 2200 ± 5 g. *n* = 4 should be big enough to pick 10 g difference between different brands. (Type I error, 0.05 and power = 0.8) *N* = 5 should be big enough to pick 12 g (SD = 5) change before and after storage. *N* = 3 should be enough to pick 20 g (SD = 5) change before and after storage.

Two hundred sixty-one CAPD patients followed up in our center were enrolled in the study. The study was performed in accordance with the Declaration of Helsinki. The study got the ethics approval by Shanghai Jiaotong University School of medicine, Renji Hospital Ethics Committee (2018)078. Written informed consent were get from each participate. All patients were on lactate buffered dextrose only solution. They were going through their routine dialysis adequacy test. Specific gravity of the drainage dialysate was measured by weighting 1 ml of the mixed 24 h drained dialysate, the same sample as their dialysis adequacy test. Specific gravity of pure water was also measured by the same method to serve as control. Dialysate sodium, potassium, protein and glucose were also measured in the mixed 24 h drained dialysate.

### Statistical analysis

One way ANOVA was used to measure the difference between different brands. General linear model were used for repeated measurement of dialysate weight. One sample t test was used to clarify the difference between dialysate specific gravity and water. The correlation between specific gravity and other parameter were identified by Pearson correlation. IBM SPSS statistics 20 was the software used for the study.

## Results

### Dialysate bags of different brand weight different at baseline

There was significant difference in weight between the four different brands. The weight was from 2221.9 ± 1.9 g to 2261 ± 3.7 g for the whole 2 L bag with outer package (*P* < 0.01). The outer package itself was different in weight too. It was between 19.1 ± 0.4 g to 21.6 ± 0.3 g (*P* < 0.01). But the big weight difference of whole dialysate bag could not be explained by the weight difference in outer package (Table 2).

**Table 2:** Dialysate weight at baseline in different brand

	whole bag	outer package	bag without outer package
A(*n* = 4)	2250 ± 3.6	19.7 ± 0.1	2230.3 ± 3.5
B(*n* = 4)	2221.9 ± 1.9	21.6 ± 0.3	2200.3 ± 1.8
C(*n* = 4)	2261.2 ± 3.7	20.4 ± 0.2	2240.8 ± 3.6
D(*n* = 4)	2229.8 ± 2.1	19.1 ± 0.4	2210.7 ± 2.1
*P* value	< 0.01	< 0.01	< 0.01

### Dialysate bags lost weight over 12 months of storage

#### Storage duration and condition had impact on weight loss

Over the 12 months of storage, all dialysate bags lost weight. The dialysate bags lost more weight at 12 months compared to 6 months (*P* < 0.01, Table 2). The higher temperature and lower humidity storage condition was related to more significant weight loss (Table 3).

**Table 3:** Dialysate weight at baseline, 6 months and 12 months in different conditions

	baseline	6 months	12 months	*P* value
condition 1(*n* = 19)	2238.9 ± 16.5	2238.6 ± 16.5	2238.3 ± 16.6	< 0.01
condition 2 (*n* = 18)	2238.6 ± 14.9	2230.2 ± 15.2	2224.8 ± 15.8	< 0.01
condition 3 (*n* = 18)	2239.1 ± 13.7	2225.6 ± 14.2	2217.2 ± 15.3	< 0.01
condition 4 (*n* = 19)	2240 ± 16	2202.9 ± 21.3	2178.3 ± 26.7	< 0.01

#### Dialysate with PVC package lost more weight than non-PVC package

**Fig. 1:**
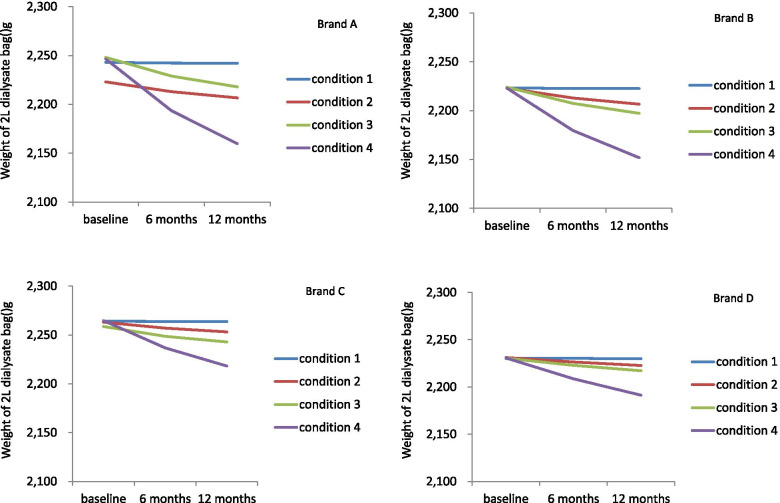
Weight loss in each brand over 12 months storage in different condition. Over the 12 month’s storage, dialysate bags lost significant weight. The weight loss was greater in higher temperature and lower humidity. PVC package was related to more significant weight loss over 12 month’s storage. Brand A and brand B were in PVC package. Brand C and brand D were in non-PVC package

The weight loss of each brand over 12 month’s storage was shown in Fig. 1 and Additional file 1: Table 1. The weight of dialysate bag at 12 months depended on baseline weight, storage condition and package type (PVC or non-PVC). PVC package was related to greater weight loss over 12 month’s storage. Generalized linear model were displayed in Table 4.

**Table 4:** generalized linear model of dialysate weight at 12 months of storage

	Multivariate		
	Coeff.	95% CI	***P***
baseline weight	.871	0.75,0.99	< 0.01
condition 1	60.975	56.21,65.74	< 0.01
condition 2	48.131	43.3,52.97	< 0.01
condition 3	39.756	34.85,44.66	< 0.01
condition 4(ref)	0		
non-PVC	16.296	12.69,19.91	< 0.01
PVC(ref)	0		
intercept	220.28	− 49.2489.8	

The equation of dialysate bag weight according to the generalized linear model can be expressed as following:

$$\mathsf{Weight}\ \mathsf{at}\ \mathsf{12}\ \mathsf{months}\ \left[\mathsf{condition}\left({\mathsf{i}}\right),\mathsf{PVC}\left({\mathsf{j}}\right)\right]=\mathsf{0.871}\ast \mathsf{Baseline}\ \mathsf{weight}+\mathsf{condition}\left(\mathsf{i}\right)+\mathsf{PVC}\left(\mathsf{j}\right)+\mathsf{220.28}\ \left(\mathsf{i}\mathsf{ntercept}\right)$$

#### The specific gravity of dialysate drainage was significantly higher than water and it was related to dialysate protein concentration

The specific gravity of dialysate drainage was 1.0136 ± 0.009 g/l, which was significantly higher than pure water (*n* = 261, *P* < 0.01). All patients enrolled were on manual exchange and on traditional lactate buffered dextrose solution. The correlation between specific gravity and dialysate protein concentration was significant (*r* = 0.139, *P* = 0.024) (Table 5).

**Table 5:** correlation between specific gravity and other solute concentration

	specific gravity	potassium	sodium	chlorine	glucose	protein
Mean ± SD	1.0136 ± 0.009 (g/l)	3.35 ± 0.67 (mmol/L)	136.14 ± 4.5 (mmol/L)	99.65 ± 5.22 (mmol/L)	30.54 ± 9.84 (mmol/L)	664.74 ± 280.03 (mg/mL)
specific gravity	1	.064	.113	−.037	−.011	.139*
potassium	.064	1	.157*	.261**	−.344**	.254**
sodium	.113	.157*	1	.680**	−.328**	.270**
chlorine	−.037	.261**	.680**	1	−.450**	.346**
glucose	−.011	−.344**	−.328**	−.450**	1	−.368**
protein	.139*	.254**	.270**	.346**	−.368**	1

* *P* < 0.05, **P < 0.01, SD, standard deviation

#### The size of potential measurement misleading of weighting the drained bag to estimate UF in clinical practice

Taking the average specific gravity from our dextrose only cohort (1.0136 g/ml), the potential over estimation of UF in a CAPD patient with 8 L input volume and 1 L UF was calculated as following.

Reported UF (L, misleading by kg)

$$=\left[\mathsf{8L}\ \left(\mathsf{input}\ \mathsf{volume}\right)+\mathsf{1L}\ \left(\mathsf{UF}\right)\right]\ast \mathsf{1.0136}\ \left(\mathsf{g}/\mathsf{ml}\right)-\mathsf{8L}\ \left(\mathsf{input}\ \mathsf{volume}\right)$$

$$=\mathsf{1.122}\left(\mathsf{L},\mathsf{misleading}\ \mathsf{by}\ \mathsf{kg}\right)\ \left(\mathsf{over}\ \mathsf{estimate}\ \mathsf{by}\ \mathsf{0.12}\mathsf{2L}\right)$$

For icodextrin, the specific gravity is even higher than dextrose solution. Icodextrin is not available in Shanghai. Prof Simon Davies shared the data in Stoke on Trent.The mean specific gravity of icodextrin long dwell was1.026 ± 0.006 g/ml. In another word, the potential over estimation of UF in CAPD for a single icodextrin dwell (2 L) with 0.4 L UF was calculated below.

Reported UF (L, misleading by kg)

$$=\left[\mathsf{2L}\ \left(\mathsf{input}\ \mathsf{volume}\right)+\mathsf{0}.\mathsf{4L}\ \left(\mathsf{UF}\right)\right]\ast \mathsf{1.026}\ \left(\mathsf{g}/\mathsf{ml}\right)-\mathsf{2L}\ \left(\mathsf{input}\ \mathsf{volume}\right)$$

$$=\mathsf{0.461}\left(\mathsf{L},\mathsf{misleading}\ \mathsf{by}\ \mathsf{kg}\right)\ \left(\mathsf{over}\ \mathsf{estimate}\ \mathsf{by}\ \mathsf{0.06}\mathsf{1L}\right)$$

## Discussion

UF is clearly important for patient survival in peritoneal dialysis. It is also an important parameter for peritoneal membrane function. Precise measurement of UF is also the base of correct estimation of other solute removal, such as sodium removal and urea and creatinine clearance.

Clinical UF measurement is different from measuring fluid volume in the lab. It should be as simple as possible for the patient to measure several times per day. It should have minimal risk to expose the patient or care giver to body fluid. Current clinical UF measurements as suggested by Bernardini J and Mahon A is weight the “whole” drained bag and minus the weight of empty bag and the expected input volume, the labeled volume plus overfill volume [5, 8]. Some carefully designed clinical trials measured dialysate bags before and after, which solve most problem of uncertain UF measurement but not all. It also mean more treatment load for patients. The current study was to understand the difference between clinical measured UF and real UF. The effect of evaporation and specific gravity in clinical UF measurement were tested in the study.

### Overfill existed in all brands, but different in each brand

We knew overfill existed in all brands. But how big the difference was was not clear to the public. Theoretically, overfill may be different between brands, type of bags and even manufacture batches. We picked the 2 L, lactate buffered, 2.5% dextrose dialysate from four different brands. It was just to get a rough idea of how big the difference was. Ideally, the manufacturer should be encouraged to publish regular audits of overfill for each type of dialysate.

### Storage condition made difference over long storage duration

As a general rule, close to room temperature (25 °C) was suggested for any medication storage if without specific instruction. However, in real life, the dialysate stored in family was likely to be in a non-air conditioned room. A wide range of storage condition was possible worldwide. According to the storage instruction of most commercial dialysate, lower than 0 degree should be avoided. For the higher limitation of storage condition, the instruction in some countries stated that more than brief exposure up to 40 °C should be avoided and recommend the product be stored at room temperature (25 °C). In some countries the instruction of dialysate did not mention it. From the current study, we definitely suggested to store dialysate bags in cool condition as far as possible. We also gave strong evidence why more than brief exposure up to 40 °C should be avoided. It could also be a problem in clinical trials. For example, in studies mean to test new PD solutions. All the new solutions for the whole study may be produced in one batch and stored for further use throughout the whole study. The study may last for 1 year. The control group, in most cases, using the commercially available solution, was likely to use the relatively fresh bags as they are continuously produced. This difference may cause systemic error. For clinical trials, weight dialysate bags before and after is strongly suggested. The fact that temperature and humidity had effect on dialysate volume may also contribute to the center effect of ultrafiltration and sodium removal in multicenter observational study or national registration study.

### PVC and non PVC package show difference in evaporation

We also noticed the different character in evaporation between PVC and non PVC package. So far, there was no clinical data on UF comparing PVC and non PVC package. An ongoing clinical trial from China may give us some useful information [13]. The problem of storage duration and difference in evaporation character should be carefully treated.

### Neglecting the effect of specific gravity leaded to overestimation of UF in CAPD

It was not surprise that the specific gravity of dialysate is slightly higher than pure water. However, it had never been estimated how big this effect was. UF in manual exchange was measured by weight and transform to volume by dividing 1 g/ml (specific gravity of pure water). While in APD, UF was directly measured in volume by the APD machine.

The study clearly demonstrated the gap between weight and volume was big enough to give systemic error when comparing UF between CAPD and APD. However, measuring dialysate volume manually was not feasible. It may cause even bigger measurement error and also increase the risk of body fluid exposure. Weight instead of volume measurement was still a reasonably way for daily practice. Mobile volume measuring tool such as flowmeter may help with this problem in clinical trial scenario.

### Limitation

In the current study, we got the dialysate bags from market. We had tried our best to get the freshest dialysate available in the market for the study. The time between manufacture to baseline measurement was still slightly different between brands (from 43 to 105 days). Evaporation process started from manufacture in principle. But the effect should be small and it is what the patients actually get in real life. Secondly, theoretically, overfill should be different between brand, type and even batch. Only one type of dialysate bags in one batch from each brand were picked in the current study. However, the study was design to establish the significant difference does exist, rather than focus on the exact figure of the difference.

There was argument that in real life dialysate was not likely to store in the extreme conditions as in the current study. Taking the fact the dialysate bags may be stored in patient’s home rather than in special medical storage, the bags were likely to be stored in a room without air condition. In many regions of the world, high room temperatures (over 30-35 °C) are not infrequent for long periods of the year.

## Conclusions

In conclusion, precise UF measurement in peritoneal dialysis is much more complicated than we thought. Storage condition and duration, as well as the type of dialysate package have significant impact in dialysate bag weight before use. Evaporation is likely to be the reason behind. The fact that specific gravity of dialysate drainage is higher than 1 g/ml overestimates UF in manual exchanges, which contributes to systemic measurement error of UF in CAPD.

## Supplementary Information

**Additional file 1: Table 1.** whole dialysate bag weight according to different brand and storage condition at different time points.

## Data Availability

The datasets used and/or analysed during the current study available from the corresponding author on reasonable request.
