# Supplementary material for: Understand the difference between clinical measured ultrafiltrationand real ultrafiltration in peritoneal dialysis
Source: BMC Nephrol. 2021 Nov 15;22:382. doi: 10.1186/s12882-021-02589-3 (PMC8594243; doi:10.1186/s12882-021-02589-3)
Supplement: Supplementary file 1 — Additional file 1: Table 1. whole dialysate bag weight according to different brand and storage condition at different time points. [file 12882_2021_2589_MOESM1_ESM.docx]

Table 1 whole dialysate bag weight according to different brand and storage condition at different time points

|  |  | n | baseline | 6 months | 12 months |
| --- | --- | --- | --- | --- | --- |
| Brand A (PVC) | condition 1 | 5 | 2243±11.9 | 2242.4±11.9 | 2242.1±11.9 |
|  | condition 2 | 5 | 2247.1±3.5 | 2235.3±3.6 | 2228±3.7 |
|  | condition 3 | 5 | 2248.1±5.3 | 2229±5.5 | 2217.9±5.7 |
|  | condition 4 | 5 | 2246.4±2.9 | 2193.4±7.1 | 2159.7±9.2 |
| Brand B (PVC) | condition 1 | 5 | 2223.3±1.3 | 2222.8±1.3 | 2222.6±1.3 |
|  | condition 2 | 5 | 2223±0.9 | 2213±1.1 | 2206.6±1.2 |
|  | condition 3 | 5 | 2224±4 | 2207.4±4.3 | 2197.3±4.5 |
|  | condition 4 | 5 | 2223.1±1.6 | 2179.7±2.1 | 2151.9±2.5 |
| Brand C (non-PVC) | condition 1 | 4 | 2264.1±5.7 | 2263.9±5.7 | 2263.8±5.7 |
|  | condition 2 | 3 | 2263±4.2 | 2257±4.1 | 2253.2±4.1 |
|  | condition 3 | 3 | 2258.6±1.8 | 2248.7±1.6 | 2242.8±1.8 |
|  | condition 4 | 4 | 2264.7±4.2 | 2236.8±4.3 | 2218.2±3.8 |
| Brand D (non-PVC) | condition 1 | 5 | 2230.1±1.3 | 2230.2±1.3 | 2229.8±1.3 |
|  | condition 2 | 5 | 2231.1±2.6 | 2226.3±2.7 | 2222.7±2.8 |
|  | condition 3 | 5 | 2230.4±1.9 | 2222.8±1.9 | 2217.2±1.9 |
|  | condition 4 | 5 | 2230.7±1.6 | 2208.6±1.1 | 2191.3±1.9 |
